# *Salmonella* osteomyelitis of the distal radius in a healthy pregnant woman

**DOI:** 10.5194/jbji-6-1-2020

**Published:** 2020-07-13

**Authors:** Akio Sakamoto, Yoshitsugu Chigusa, Takashi Noguchi, Shuichi Matsuda

**Affiliations:** 1 Department of Orthopaedic Surgery, Graduate School of Medicine, Kyoto University, Kyoto, 606-8507, Japan; 2 Department of Gynecology and Obstetrics, Graduate School of Medicine, Kyoto University, Kyoto, 606-8507, Japan

## Abstract

Although characteristic, *Salmonella* is a rare cause of osteomyelitis, especially in healthy individuals. A 25-year old primigravida at 29 weeks' gestation noticed pain and swelling in her right wrist. Her leukocyte count was normal, but her C-reactive protein level was slightly elevated, at 1.1 mg dL${}^{-1}$ (normal range, <0.2 mg dL${}^{-1}$). Plain radiography showed an osteolytic lesion in the distal radius, and magnetic resonance imaging (MRI) showed an extraosseous fluid collection with bone edema in addition to the osseous lesion. After a needle biopsy was performed, the skin overlying the lesion became ulcerated at the site of the needle tract. We drained whitish pus from the site; both this pus and the original biopsy specimen grew *Salmonella* on culture. We diagnosed *Salmonella* osteomyelitis and began intravenous antibiotic therapy, avoiding oral quinolones to prevent fetotoxicity. Her symptoms resolved, as did the bone edema and fluid collection. Ossification occurred at the site of osteolysis, with localized abnormal signal intensity persisting on MRI. This rare case of *Salmonella* osteomyelitis was treated without surgery; the patient's pregnancy influenced the treatment course.

## 1 Introduction

*Salmonella* infections are associated with gastroenteritis, enteric fever, bacteremia, focal infection (e.g., soft tissue), and a chronic carrier state (Cohen et al., 1987). Osteomyelitis is a rare complication of *Salmonella* infection, reportedly occurring in about 0.8 % of infections (Cohen et al., 1987; McAnearney and McCall, 2015). Patients with *Salmonella* osteomyelitis usually have immunosuppressive conditions, including sickle cell anemia, hemoglobinopathy, or diabetes mellitus (Banky et al., 2002; Pak and Pham, 2017). *Salmonella* osteomyelitis is rare in healthy individuals (Tonogai et al., 2015). The diaphysis of the long bones (i.e., femur, humerus) is most commonly affected (Declerq et al., 1994). Reports of other affected bones have included the lumbar vertebrae, radius, ulna, and tibia. *Salmonella* osteomyelitis reportedly has a female predominance (Schneider et al., 2009).

We report herein a healthy pregnant patient with *Salmonella* osteomyelitis affecting the radius. We treated her infection with antibiotic medication but without surgery. We considered her pregnancy when determining the appropriate treatment. This case report has been approved by the institutional review board (R2499).

## 2 Case presentation

A 25-year old primigravida at 29 weeks' gestation noticed pain and swelling in her right wrist; these symptoms worsened rapidly over the course of 2 months. Initially, she thought she had tendinitis of the wrist. She reported no fever and no general fatigue. Her medical history was significant for smoking 20 cigarettes per day before her pregnancy. She was an office worker and had no chance to handle plants or animals; she kept no pets. She had no history of gastroenteritis to suggest *Salmonella* infection, nor did she have a history of sickle cell disease or immunodeficiency. She did have a history of cervical intraepithelial neoplasia associated with human papillomavirus infection and a benign cystic mass of the right ovary that was under observation. She had no history of trauma to the wrist or bone infection of the distal radius.

**Figure 1:**
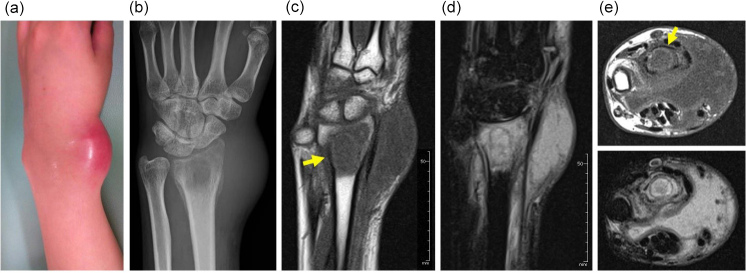
Swelling and redness are seen at the wrist **(a)**. Plain radiography shows an osteolytic lesion in the distal radius with soft tissue swelling **(b)**. Magnetic resonance imaging shows low signal intensity on T1-weighted imaging **(c)** and high signal intensity on T2-weighted, fat-suppressed imaging **(d)** in both the osteolytic area and the surrounding bone marrow. A fluid collection is seen (**e** – top image, T1-weighted imaging; **e** – bottom image, T2-weighted imaging). Yellow arrows point to a rim of high signal intensity on T1-weighted imaging (“penumbra sign”) (**c**, **e** – top image).

She visited a nearby hospital and was referred to our institution with a working diagnosis of malignancy because of the rapidly progressive symptoms. On physical examination at our hospital, her wrist was swollen and the overlying skin was reddish and taut (Fig. 1a). All values of the laboratory analysis were normal except for the inflammatory marker C-reactive protein, which was slightly elevated at 1.1 mg dL${}^{-1}$ (normal range, <0.2 mg dL${}^{-1}$). Her leukocyte count was also elevated at 12 810 mm${}^{-3}$ (normal range, 2900–9100 mm${}^{-3}$). The differential revealed neutrophils, 76.1 % (normal range, 46 %–62 %); lymphocytes, 17.3 % (normal range, 30 %–40 %); monocytes, 5.5 % (normal range, 4 %–7 %); eosinophils, 0.8 % (normal range, 3 %–5 %); and basophils, 0.3 % (normal range, <1 %).

Plain radiography revealed an ill-defined osteolytic lesion in the radius (Fig. 1b). Magnetic resonance imaging (MRI) showed that the lesion had homogenous low-signal intensity on T1-weighted images and high signal intensity on T2-weighted images; the involved area was larger than the osteolytic area seen on plain radiography. Within the area of high signal intensity, an elliptical line of low signal intensity was observed. Inside this ellipse, a rim of high signal intensity was noted; this rim also had high signal intensity on T1-weighted imaging, consistent with the “penumbra sign”, characteristic of subacute osteomyelitis (Grey et al., 1998). The presence of a fluid collection in the soft tissue anterior to the distal radius, with low signal intensity on T1-weighted imaging and high signal intensity on T2-weighted imaging, suggested an abscess (Fig. 1c–e). The diagnosis based on imaging findings was osteomyelitis. However, secondary infection of a malignant bone tumor of Ewing's sarcoma was on our differential diagnosis.

A needle biopsy specimen was obtained from the radius underlying the area of swelling, and pathologic examination excluded the presence of a neoplastic lesion. The diagnosis, based on her symptoms and the elevated inflammatory markers, was osteomyelitis of the radius. While waiting for pathologic confirmation of the diagnosis, we administered intravenous ceftriaxone, 1 g daily, for nonspecific myelitis.

Culture of the fluid collection overlying the radius revealed *Salmonella*, but blood culture failed to grow any bacteria. Unfortunately, the species of *Salmonella* was not determined because the reported diagnosis was not enteritis. Histologic examination of the needle biopsy specimen showed several types of inflammatory cells: aggregates of neutrophils and plasmacytes. The histologic diagnosis was infection, not neoplasm, and the final diagnosis was *Salmonella* osteomyelitis. The microbiological susceptibility panel was available on the fifth day of antibiotic therapy (Table 1). The patient was continued on intravenous ceftriaxone, but the dose was increased to 2 g daily for 6 weeks, until she reached 35 weeks' gestation.

**Table 1:** Microbiologic susceptibility panel.

Antibiotic	Minimum inhibitory
	concentration
Ampicillin	≤4.0
Piperacillin	≤8.0
Ampicillin/sulbactam	≤8.0
Amoxicillin/clavulanic acid	≤4.0
Tazobactam/piperacillin	≤8.0
Cefazolin	≤4.0
Cefaclor	≤8.0
Cefpodoxime	≤1.0
Cefotiam	≤8.0
Cefotaxime	≤1.0
Ceftriaxone	≤1.0
Sulbactam/cefoperazone	≤16.0
Cefdinir	≤0.5
Cefepime	1.0
Ceftriaxone	≤4.0
Cefepime	≤2.0
Cefozopran	≤4.0
Cefmetazole	≤16.0
Flomoxef	≤4.0
Azithromycin	≤4.0
Imipenem	≤1.0
Meropenem	≤1.0
Doripenem	≤1.0
Ciprofloxacin	≤0.25
Levofloxacin	≤0.5
Sitafloxacin	≤1.0
Gentamicin	≤2.0
Tobramycin	≤4.0
Amikacin	≤4.0
Minocycline	≤2.0
Fosfomycin	≤4.0
Sulfamethoxazole/trimethoprim	≤2.0

We continued to observe the patient closely, to be sure that her clinical course continued to follow a typical trajectory of infection and that we did not miss sampling any neoplastic tissue in the bone. The skin overlying the lesion became ulcerated at the site of the needle biopsy tract, and whitish pus was drained. Culture of the pus revealed *Salmonella*. The soft tissue swelling promptly resolved after the initiation of antibiotic therapy. One week after starting antibiotic treatment, the leukocyte count normalized to 6540 mm${}^{-3}$ (neutrophils, 65.0 %; lymphocytes, 27.1 %; monocytes, 6.4 %; eosinophils, 1.1 %; basophils, 1.0 %). The C-reactive protein level decreased to a slightly elevated value of 0.4 mg dL${}^{-1}$. On initial assessment, we thought that surgical curettage and drainage might be required. However, we decided not to perform surgery because of her clinical and laboratory improvement. We continued intravenous ceftriaxone and avoided oral quinolone medication to prevent fetotoxicity. We followed the patient with laboratory analysis, including C-reactive protein, once monthly. By 1 month after finishing the antibiotic course, the patient's C-reactive protein value was normal, at less than 0.1 mg dL${}^{-1}$.

We were concerned that the infection could spread to the uterus and lead to premature labor, so the patient was seen every 1 to 2 weeks by obstetricians during the treatment period, and the fetus was evaluated by ultrasonography. At 40 weeks' gestation, the patient underwent emergency cesarean delivery for a nonreassuring fetal heart rate pattern. She was delivered of a male infant weighing 3168 g. The neonate was examined by neonatologists immediately after birth and 1 month later; his course was favorable. The patient's ovarian mass was resected at the time of cesarean delivery and found to be a cystic lesion, 5×4 cm, containing hair and adipose tissue. Histologic examination revealed squamous epithelium and skin appendages, leading to the diagnosis of a mature teratoma, or dermoid cyst.

**Figure 2:**
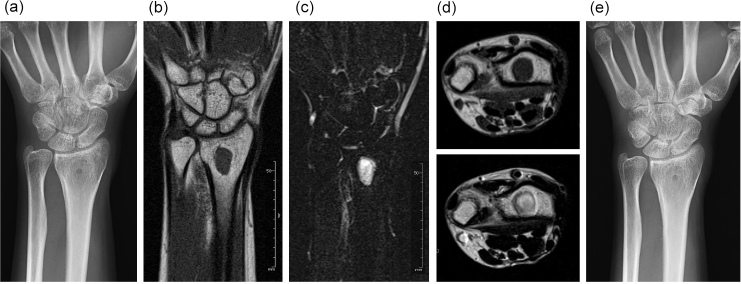
One year after treatment, the previously osteolytic area is ossified **(a)**. The lesion has homogenous, low signal intensity on T1-weighted imaging **(b)** and homogenous, high signal intensity on T2-weighted, fat-suppressed imaging **(c)**. No fluid collection is present (**d** – top image, T1-weighted imaging; **d** – bottom image, T2-weighted imaging). Two years after treatment, no recurrence is seen **(e)**.

We followed the patient with plain radiography every 3 months for 1 year and then every 6 months for several years. We obtained an MRI once or twice yearly for several years. The osteolytic area became ossified, as seen on plain radiography performed 1 year after treatment (Fig. 2a). At around this time, MRI showed persistent low signal intensity on T1-weighted imaging and high signal intensity on T2-weighted imaging, without any abnormal signal indicating edema of the bone marrow. The fluid collection had resolved (Fig. 2b–d). There was no recurrent infection observed 24 months after completing antibiotic therapy (Fig. 2e).

## 3 Discussion

Immunologic changes occur during pregnancy to achieve a careful balance between tolerance of fetal antigens and continued immunity against infectious agents (Fuhler, 2020). However, pregnant women are reportedly 17 times more likely to be affected by the foodborne Gram-positive bacterium *Listeria monocytogenes*, which can cause premature delivery, miscarriage, and stillbirth (Pohl et al., 2019). There is only a single available case report of *Salmonella* osteomyelitis during pregnancy (Agustsson et al., 2009), so it seems safe to assume that our patient's rare osteomyelitis is attributable to pregnancy, even though pregnancy can cause immunosuppression. A plausible reason for our patient's contraction of *Salmonella* osteomyelitis remains unknown. There are three common strains of *Salmonella* that cause osteomyelitis: *S typhimurium, S typhi,* and *S enteritidis. *The only strain known to be transmitted from human to human is *S typhi* (Arora et al., 2003). Osteomyelitis caused by *S panama* has also been reported in the literature (van Cappelle et al., 1995). Unfortunately, we were not able to determine the specific species that caused our patient's osteomyelitis.

The reported symptoms of *Salmonella* osteomyelitis are pain and various degrees of swelling. These symptoms are not limited to bone infection caused by *Salmonella,* and they do not always occur with *Salmonella* infection. When present, the duration of symptoms ranges from a few months to several years (Declerq et al., 1994). Our patient experienced acute pain and swelling, but her only elevated inflammatory marker was C-reactive protein. The osteolytic condition of the radius suggested a subacute or chronic condition. The logical diagnosis was therefore subacute osteomyelitis or chronic osteomyelitis with acute worsening.

Although our patient's MRI findings suggested osteomyelitis with a pus collection, the osteolytic lesion meant that the differential diagnosis included a neoplastic lesion with secondary infection. A transitional zone with relatively high signal intensity was noted on T1-weighted imaging, located between the abscess and the sclerotic bone marrow. This sign, called the “penumbra sign” (Grey et al., 1998), is characteristic of subacute osteomyelitis and suggests infection rather than neoplasm. Biopsy was useful for ruling out a neoplastic lesion. A culture was necessary to determine the proper treatment, because *Salmonella* osteomyelitis is indistinguishable from other etiologies, such as pyogenic or tubercular osteomyelitis, in terms of symptoms and imaging findings (Arora et al., 2003).

Chronic and subacute osteomyelitis are both conventionally treated by surgical debridement combined with antibiotic therapy (van Cappelle et al., 1995). Typically, surgery is performed first, followed by antibiotic treatment (Hashimoto et al., 2018). In chronic osteomyelitis, radical debridement is recommended (Carlson et al., 1994). In our patient with acute-onset osteomyelitis, pus was drained through ulcerated skin. For such cases, a combination of intravenous and oral antibiotics is usually used (Hashimoto et al., 2018). Third-generation cephalosporins and new oral quinolones are reportedly useful for *Salmonella* osteomyelitis (Banky et al., 2002). Our patient was given only intravenous ceftriaxone, to avoid the possible fetotoxic effects of oral quinolones.

## 4 Conclusions

We report herein the case of a pregnant woman with *Salmonella* osteomyelitis of the radius. Treatment decisions in her case required special attention, given her pregnancy. We drained the pus from her radial lesion but did not perform surgical debridement. We used intravenous antibiotic medication that was safe for pregnancy.

Learning points

- *Salmonella* is a rare cause of osteomyelitis, especially in healthy individuals.
- *Salmonella* osteomyelitis may be treated with intravenous antibiotic medication, avoiding surgery.
- Oral quinolones are avoided to prevent fetotoxicity.

## Data Availability

No data sets were used in this article.
